# Child immunization status according to number of siblings and birth order in 85 low- and middle-income countries: a cross-sectional study

**DOI:** 10.1016/j.eclinm.2024.102547

**Published:** 2024-03-16

**Authors:** Francine S. Costa, Larissa A.N. Silva, Bianca O. Cata-Preta, Thiago M. Santos, Leonardo Z. Ferreira, Tewodaj Mengistu, Daniel R. Hogan, Aluisio J.D. Barros, Cesar G. Victora

**Affiliations:** a International Center for Equity in Health, Federal University of Pelotas, Pelotas, Brazil; b Postgraduate Program in Epidemiology, Federal University of Pelotas, Pelotas, Brazil; c Public Health Department, Federal University of Parana, Curitiba, Brazil; d Gavi, The Vaccine Alliance, Geneva, Switzerland

**Keywords:** Vaccination, Siblings, Birth order, Health surveys, Global health

## Abstract

**Background:**

Identification of unvaccinated children is important for preventing deaths due to infections. Number of siblings and birth order have been postulated as risk factors for zero-dose prevalence.

**Methods:**

We analysed nationally representative cross-sectional surveys from 85 low and middle-income countries (2010–2020) with information on immunisation status of children aged 12–35 months. Zero-dose prevalence was defined as the failure to receive any doses of DPT (diphtheria-pertussis-tetanus) vaccine. We examined associations with birth order and the number of siblings, adjusting for child's sex, maternal age and education, household wealth quintiles and place of residence. Poisson regression was used to calculate zero-dose prevalence ratios.

**Findings:**

We studied 375,548 children, of whom 13.7% (n = 51,450) were classified as zero-dose. Prevalence increased monotonically with birth order and with the number of siblings, with prevalence increasing from 11.0% for firstborn children to 17.1% for birth order 5 or higher, and from 10.5% for children with no siblings to 17.2% for those with four or more siblings. Adjustment for confounders attenuated but did not eliminate these associations. The number of siblings remained as a strong risk factor when adjusted for confounders and birth order, but the reverse was not observed. Among children with the same number of siblings, there was no clear pattern in zero-dose prevalence by birth order; for instance, among children with two siblings, the prevalence was 13.0%, 14.7%, and 13.3% for firstborn, second, and third-born, respectively. Similar results were observed for girls and boys. 9513 families had two children aged 12–35 months. When the younger sibling was unvaccinated, 61.9% of the older siblings were also unvaccinated. On the other hand, when the younger sibling was vaccinated, only 5.9% of the older siblings were unvaccinated.

**Interpretation:**

The number of siblings is a better predictor than birth order in identifying children to be targeted by immunization campaigns. Zero-dose children tend to be clustered within families.

**Funding:**

10.13039/100001125Gavi, the Vaccine Alliance.

**Research in context**

**Evidence before this study**

Identifying factors that drive child vaccination is crucial for safeguarding children from infectious diseases. We reviewed evidence on the impact of birth order and number of siblings on vaccination status in low and middle-income countries (LMICs) through a systematic search of PubMed, Embase, and Web of Science up to September 10, 2023. Out of 2200 articles found, 31 addressed vaccination in LMICs by birth order and/or number of siblings (see Supplemental Box 1). Although results varied across studies, most found that later birth order and a greater number of siblings are associated with incomplete vaccination or non-vaccination.

**Added value of this study**

Few studies had simultaneously investigated the roles of birth order and number of siblings. In the largest set of analyses on this topic, we studied approximately 400,000 children from 85 low and middle-income countries. Zero-dose prevalence was more consistently associated with larger numbers of siblings than with a child's birth order. In families with more than one child, there was strong intra-familial clustering of zero-dose children.

**Implications of all the available evidence**

Whereas most of the available evidence comes from single-country studies of immunisation coverage according to birth order, our findings are derived from a multi-country analysis, suggesting the number of children in a family is more strongly predictive of zero-dose children. In order to reduce morbidity and mortality due to infectious diseases, large families should receive special attention during immunization efforts, and siblings of unvaccinated children should be actively traced regardless of their age.

## Introduction

The role of vaccination in safeguarding global public health cannot be understated. Vaccines stand as a pivotal and cost-effective health intervention, recognised for their ability to prevent and even potentially eradicate diseases.^1^^,^^2^ To ensure that no child is left behind, efforts within the immunisation community have been recently focused on identifying and reaching children without any routine vaccinations, proxied by children who have not received any doses of diphtheria-pertussis-tetanus (DPT) containing vaccines, commonly referred to as “zero-dose children”. These children may be identified through household surveys as well as by leveraging data from administrative health information systems. The terms “zero-dose” and “zero-DPT” are widely utilized as synonyms.^3^, ^4^, ^5^, ^6^, ^7^, ^8^ Evaluating the prevalence of these children is instrumental in directing immunisation efforts to bridge the immunisation gap, and it is estimated by over 14 million children, or one in ten worldwide, fell into this category in 2022.^9^ As part of the World Health Organization (WHO) Immunisation Agenda 2030 (IA2030), the aim of achieving a 50% reduction in zero-dose prevalence by 2030 has been set.^2^

Zero-dose children tend to be disproportionately concentrated in regions affected by conflict and humanitarian emergencies, remote rural areas, and among families living in poverty.^10^, ^11^, ^12^, ^13^ Such disparities in immunisation coverage have been the subject of comprehensive analyses in numerous low- and middle-income countries (LMICs). These studies have identified influential factors affecting immunisation rates, including household wealth, maternal schooling, and place of residence.^14^, ^15^, ^16^, ^17^, ^18^ Additionally, studies have identified ethnicity^19^^,^^20^ and religion^20^ as determinants of vaccine coverage, albeit with variations between countries. Conversely, the sex of the child has shown minimal association with vaccination status in the majority of countries studied.^14^^,^^15^^,^^21^

We located 31 published studies on potential associations between birth order and immunisation status using survey data, and six studies for the association with number of siblings or family size (Supplemental Panel 1). Most studies suggest that first-born children are more likely to be immunised than children with later birth orders, and that the number of siblings was inversely associated with immunisation coverage. Only two studies included analyses for more than one LMIC country,^22^^,^^23^ and three provided analyses in which birth order was adjusted for family size and vice-versa.^22^^,^^24^^,^^25^

In the present study, we analysed data from 85 LMICs to investigate how zero-dose prevalence varies relative to birth order and the number of siblings. We also explored the possibility of confounding by known drivers of immunisation coverage and examined whether the child's sex might modify these associations. Lastly, we explored the possibility of familial clustering of zero-dose children.

## Methods

### Study design

We analysed secondary data from nationally representative cross-sectional surveys carried out in low- and middle-income countries. STROBE (*Strengthening the Reporting of Observational studies in Epidemiology*)^26^ recommendations were applied to study's report.

### Ethics

Ethical clearance was the responsibility of the institutions that administered the surveys and all analyses relied on anonymised databases.

### Procedures

The database of the International Centre for Equity in Health at the Federal University of Pelotas includes publicly available datasets from nationally representative Demographic and Health Surveys (DHS) and Multiple Indicator Cluster Surveys (MICS).^27^^,^^28^ We screened over 450 surveys from 125 countries to select the most recent survey from each country carried out since 2010 with information on immunisation status of children aged 12–35 months. Both types of surveys are highly comparable in terms of sampling design and indicator definitions.^29^ Surveys from 85 countries (47 DHS and 38 MICS) had information on number of siblings and birth order (Supplemental Tables S1 and S2).

### Outcomes and explanatory variables

The outcome under study is zero-dose prevalence, defined as those who failed to receive any dose of a DPT-containing vaccine, as recommended by international guidelines.^30^ The standard age range of 12–23 months for vaccine coverage analyses was extended to increase the number of children with younger siblings—as most surveys collect data on children aged up to 35 months. The vaccination card was the primary source of information, supplemented by maternal recall when a card was not available. Children without any information on vaccination status were considered as unvaccinated as recommended by WHO.^30^ Missing values accounted for 2.9% of the total number of children and there was little variability in missingness according to birth order, number of siblings or wealth quintiles (Supplemental Table S3).

The explanatory variables were birth order and number of siblings. Birth order was coded as 1 (firstborn), 2, 3, 4 or ≥ 5. Stillbirths were not counted, and twins were considered as one birth. In DHS, the birth order variable is available in the child dataset. For MICS, it had to be derived from the birth history dataset by ordering the offspring of the mother according to their dates of birth. The number of siblings was coded as 0, 1, 2, 3 or ≥ 4. Liveborn siblings who died before the survey were included in the main analyses because these children might have still been alive at the time of vaccination of the index child. Twins or triplets were counted as two or three siblings. Sensitivity analyses restricted to surviving siblings are shown in the Supplemental Materials.

The following variables were considered as confounders given their potential associations with immunisation status and with the exposure variables: maternal age at the birth of the child (15–17 years; 18–19 years; 20–34 years; 35–49 years, household wealth quintile, maternal schooling, area of residence coded as urban or rural according to country-specific delimitations, family type, and type of information on immunisation (vaccine card or maternal recall). Household wealth quintiles were derived from household asset indices provided in the DHS and MICS datasets. These were obtained using principal component analyses of household assets and characteristics of the building, presence of electricity, water supply and sanitary facilities, among other variables associated with wealth. Because relevant assets may vary in urban and rural households, principal component analyses are initially carried out separately for each area and later combined into a single score using a scaling procedure to make area scores comparable.^31^ Maternal schooling was analysed in three categories, as reported by the survey respondent: none (no formal schooling), primary (any primary schooling, including completed primary schooling), and secondary or higher (any secondary schooling, including completed secondary schooling and partial or full higher schooling). Additionally, family type was defined as nuclear or extended. While the nuclear family is composed only of parents and their children, an extended family expands beyond this structure to encompass grandparents, other relatives, and non-relatives.^32^

Sex of the child (defined as male or female) was obtained through maternal report during the interview and was also included as a covariate. The frequencies of missing values for the above variables ranged from 0.0% for child sex to 1.8% for birth order (Supplemental Table S4).

### Statistics

Analyses were carried out at ecological and individual levels. The 85 countries were the units of the ecological analyses. Linear regression and Pearson's correlation were used to study the associations between zero-dose prevalence and the national average values of birth order and number of siblings. National per capita gross domestic product (GDP in US$–United States dollars) (source: The World Bank^33^) was included as a potential confounder as were maternal schooling (percent of women with no education or incomplete primary education), and percent rural population; the latter two indicators were directly calculated from the national surveys used in the analyses. Household wealth was not included because quintiles are country specific.

The individual-level analyses included all children aged 12–35 months from the 85 countries, whose data was pooled in a single dataset. Zero-dose prevalence for the possible combinations of birth order and siblings were estimated using the “margins” command in Stata, which includes an interaction between the two variables, adjusting fixed effects for country. Three regression models are presented. The first included fixed effects for country, the second also included the confounding variables listed above, and the third included confounders and both exposure variables simultaneously. Analyses of each survey dataset accounted for the multi-stage survey design and sampling weights. Because only 2.5% of children had another sibling in the analyses, we opted not to use multilevel models.

The pooled analyses included Poisson regression with robust variance to calculate prevalence ratios (PR) and 95% confidence intervals (95% CI) for each category of the explanatory variable, which are easier to interpret than odds ratios derived through logistic regression.^34^ Sensitivity analyses were carried out by restricting the sample to children aged 12–23 months, to those aged 24–35 months, and to countries from Sub-Saharan Africa.

Intrafamilial clustering of zero-dose children was assessed using the command “margins”, investigating zero-dose status within families with a child aged 12–23 months and another child aged 24–35 months, adjusted to fixed effects for country. The kappa statistic was calculated for assessing the probability of agreement.

Both ecological and individual level analyses were weighted by the populations of children aged 12–35 months in each country as of 2017 (median year of all surveys), according to the World Bank.^35^

The analyses were carried out with Stata (StataCorp. 2019. Stata Statistical Software: Release 17. College Station, TX: StataCorp) and R (R Core Team, 2020, version 4.1.0. R Foundation for Statistical Computing, Vienna, Austria).

### Role of funding source

Authors TM and DRH are employed by GAVI, the funder Institution of the study. As authors, they have participated in the study design, interpretation of results and writing up of the manuscript.

## Results

Our analyses included 375,548 children from 85 countries. Supplemental Table S1 shows the list of countries and surveys. The regions with the largest number of countries were West & Central Africa (22 countries) and Eastern & Southern Africa (18 countries), followed by East Asia & the Pacific (13 countries), Latin America & Caribbean (11 countries), Middle East & North Africa (8 countries), Eastern Europe & Central Asia (7 countries), and South Asia (6 countries). Supplemental Table S2 shows that 29% of the surveys were carried out in 2010–2015, 65% in 2016–2019 and 6% in 2020–2021. Just over three quarters of the children in the analyses were seen in 2016–2019. The study sample covered 88.2% of all low-income countries, 68.1% of all lower-middle income and 41.1% of all upper-middle income countries in the world. The lower participation of upper-middle income countries is due to the fact that such countries tend to monitor coverage through routine health information systems rather than surveys.^7^

The supplemental material presents information on the distribution of the sample according to exposures and confounding variables (Supplemental Table S4), on missing values (Supplemental Table S3), and on the role of maternal recall in providing immunisation information (Supplemental Table S5).

As expected, ecological analyses at national level showed that mean birth order was strongly correlated with mean number of siblings (Pearson r = 0.994; 95% CI 0.991–0.996; p-value<0.001) (Supplemental Figure S1). Country by country results are presented in Supplemental Table S6 with correlation coefficients ranging from 0.838 in Yemen to 0.950 in Bangladesh.

The median national zero-dose prevalence was 8.1% (Interquartile Range [IQR] 4.4%; 18.2%). The mean national zero-dose prevalence was inversely correlated to per capita GDP (Pearson r = −0.332; 95% CI −0.509 to −0.128; p-value<0.001) (Supplemental Figure S2). Zero-dose prevalence increased 11.1 (95% CI 8.5; 13.6) percent points per unit increase in the national mean number of siblings; after adjusting for national GDP, maternal schooling and rural population, the association was attenuated (6.3 percent points increase; 95% CI 2.2; 10.4). Zero-dose prevalence increased 11.6 (95% CI 9.0; 14.2) percent points per unit increase in the national mean birth order in the unadjusted analysis and by 6.9 (95% CI 2.7; 11.2) percent points after adjustment. Supplemental Figures S3 and S5 show the unadjusted scatter diagrams while Supplemental Figures S4 and S6 show the adjusted results.

Individual-level analyses were carried out after pooling survey data from all countries. Almost one quarter (23.7%) of the children did not have any siblings; 29.2% had one, 17.5% two, 10.9% three and 18.7% had four or more siblings. The birth order distribution was as follows: 29.9% firstborn, 26.7% second, 16.2% third, 10.1% fourth and 17.0% with fifth or later birth order. Of the total, 13.7% of the children were classified as zero dose.

**Fig. 1:**
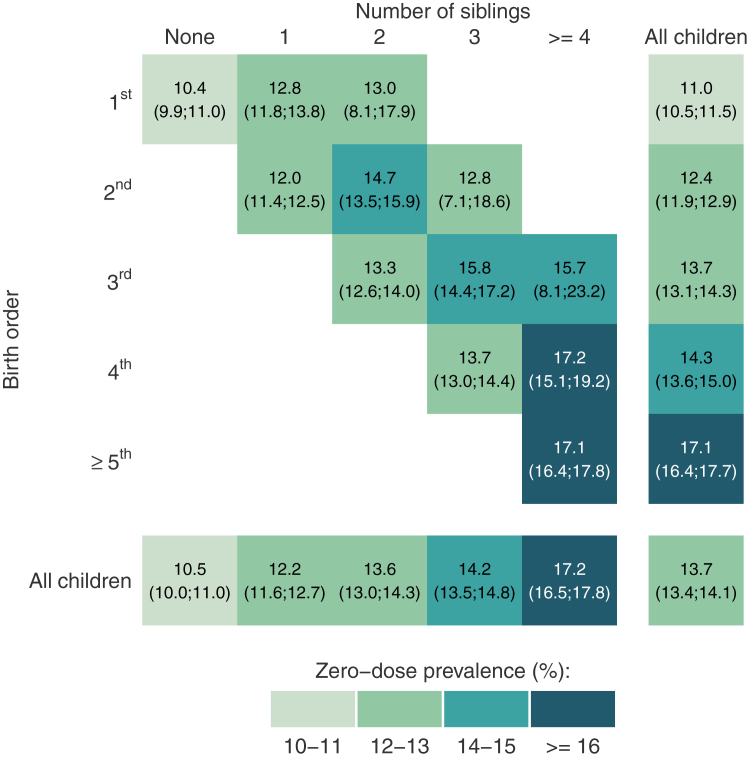
Zero-dose prevalence according to combinations of number of siblings and birth order. Pooled analyses from 85 countries. Cells with fewer than 50 children are omitted. Results were obtained analysing 85 countries with data on number of siblings and birth order. Results are adjusted using fixed effects for country. 95% confidence intervals shown in brackets.

Table 1 shows that, as expected, birth order is mostly consistent with the number of siblings. A few index children (aged 12–35 months) with low birth orders had three or more siblings. This could be due to multiple pregnancies after the index child was born or to errors in the survey database. Fig. 1 shows zero-dose prevalence according to the number of siblings and birth order in an analysis that includes their interaction term. Zero-dose prevalence increased monotonically with the number of children in the household, as well as with birth order. However, within groups of the number of siblings there was no clear pattern in zero-dose prevalence by birth order, although prevalence seemed to increase and then decline with increasing birth order (note the wide confidence intervals). Within the same birth order, prevalence tended to increase with the number of siblings. There was no evidence that sex of the child modified the association between zero-dose prevalence and birth order (Supplemental Table S7).

**Table 1:** Distribution of the sample according to birth order and number of siblings.

Birth order	Number of siblings					All children
	None	1	2	3	≥4	
	N (%)	N (%)	N (%)	N (%)	N (%)	
1st	83,843 (79.4)	21,299 (20.1)	540 (0.5)	29 (0.0)	2 (0.0)	105,713 (100.0)
2nd		79,747 (86.9)	12,704 (12.8)	353 (0.3)	44 (0.0)	92,848 (100.0)
3rd			52,404 (86.4)	8468 (13.2)	275 (0.4)	61,147 (100.0)
4th				33,862 (85.2)	5703 (14.8)	39,565 (100.0)
≥5th					69,671 (100.0)	69,671 (100.0)
All children	83,843 (23.7)	101,046 (29.3)	65,648 (17.5)	42,712 (10.9)	75,695 (18.6)	368,944 (100.0)

Results restricted to 85 countries with data on number of siblings and birth order. The percentages in parentheses are row percents. Results are adjusted using fixed effects for country.

Pooled analyses from 85 countries.

Poisson regression analyses are shown in Tables 2 and 3, each of which includes the three models described above. In model 1 (with fixed effects for country) zero-dose prevalence among children with four or more siblings is 64% higher than for single children. Adjustment for confounders (model 2) leads to an important attenuation in the association, although the gradual increase in prevalence with number of siblings remains. Further adjustment for birth order shows prevalence ratios that are similar to those in the unadjusted model (Table 2).

**Table 2:** Zero-dose prevalence ratios (PR) and 95% confidence intervals (CI) according to the number of siblings.

Number of siblings	Model 1	Model 2	Model 3
	PR (95% CI)	PR (95% CI)	PR (95% CI)
None	1	1	1
1	1.16 (1.09; 1.23)	1.18 (1.1; 1.23)	1.17 (1.08; 1.27)
2	1.30 (1.23; 1.38)	1.28 (1.17; 1.34)	1.34 (1.19; 1.50)
3	1.35 (1.27; 1.45)	1.27 (1.12; 1.28)	1.46 (1.26; 1.69)
≥4	1.64 (1.54; 1.74)	1.40 (1.21; 1.38)	1.65 (1.39; 1.96)
p-value	<0.001	<0.001	<0.001

Model 1—Results are adjusted using fixed effects for country.

Model 2—Also adjusted for wealth quintiles, maternal education, maternal age at birth, area of residence, family type, vaccine recall, child's age, and sex.

Model 3—Model 2 plus birth order.

Pooled analyses from 85 countries.

**Table 3:** Zero-dose prevalence ratios (PR) and 95% confidence intervals (CI) according to birth order.

Birth order	Model 1	Model 2	Model 3
	PR (95% CI)	PR (95% CI)	PR (95% CI)
1st	1	1	1
2nd	1.13 (1.07; 1.19)	1.16 (1.10; 1.22)	1.01 (0.95; 1.09)
3rd	1.25 (1.18; 1.32)	1.23 (1.15; 1.30)	0.94 (0.84; 1.06)
4th	1.30 (1.22; 1.39)	1.22 (1.14; 1.30)	0.85 (0.73; 0.99)
≥5th	1.56 (1.47; 1.65)	1.33 (1.25; 1.42)	0.85 (0.72; 1.00)
p-value	<0.001	<0.001	0.07

Model 1—Results are adjusted using fixed effects for country.

Model 2—Also adjusted for wealth quintiles, maternal education, maternal age at birth, area of residence, family type, vaccine recall, child's age, and sex.

Model 3—Model 2 plus number of siblings.

Pooled analyses from 85 countries.

A similar set of analyses for birth order is shown in Table 3. In model 1, children with 5th or later birth order show a 56% increase in zero-dose prevalence compared to firstborn children. This association is substantially attenuated with control for confounders (model 2). Additional control for the number of siblings leads to an inversion in the risk pattern, with children with 5th or later order showing a 15% reduction in prevalence compared to firstborn children.

Supplemental Table S8 shows that these dose–response patterns were present and significant both for countries with MICS and those with DHS, although the latter tended to show stronger associations. The median GDP per capita in countries with MICS and DHS were US$ 6344 [IQR 4013; 11,042] and US$3428 [IQR 2182; 7967], which may explain the different effect sizes.

Collinearity between birth order and the number of siblings must be considered when interpreting model 3 in both tables. Supplemental Figure S1 shows that Pearson's correlation coefficient for the two variables was equal to 0.994 in the ecological analyses with countries as the units. In the individual-level analyses, the coefficient was equal to 0.890 in the pooled dataset and the variance inflation factor was equal to 18.0. Country-specific Pearson correlation coefficients are presented in Supplemental Table S6: 48 countries had coefficients between 0.84 and 0.90, 36 countries from 0.90 to 0.949 and one country (Bangladesh) above 0.95. The issue of collinearity is addressed in the Discussion.

**Fig. 2:**
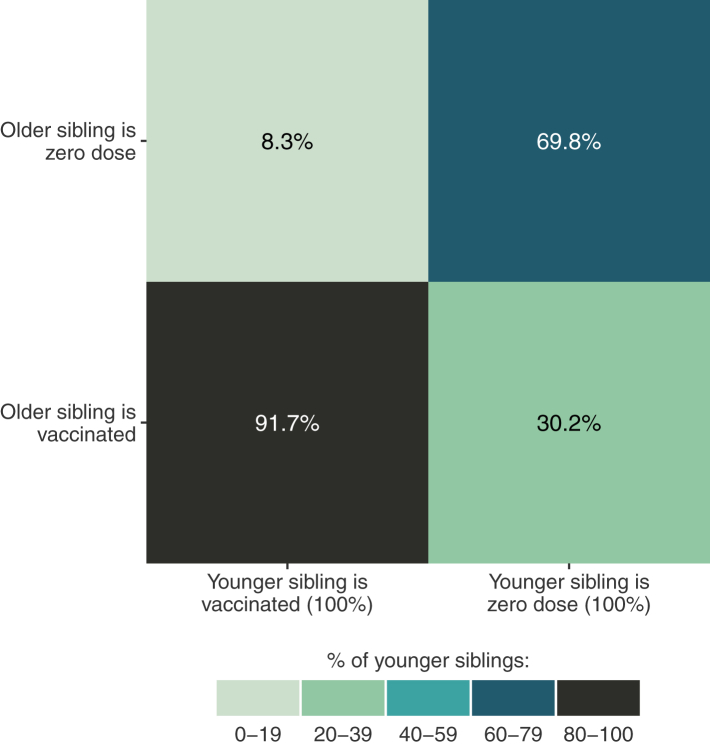
Within-family concordance of zero-dose status among 9513 pairs of siblings from 85 countries. Results are adjusted using fixed effects for country. Younger siblings are aged 12-23 months and older siblings 24-35 months.

Lastly, we assessed intrafamilial clustering of zero-dose children (Fig. 2). Our study sample included 9513 pairs of siblings from 85 countries, one of whom was aged 12–23 months and the other 24–35 months. For 88.2% of the sibling pairs, there was agreement in terms of vaccination status. The kappa statistic was equal to 0.608 (95% CI 0.588–0.628), indicating substantial agreement within pairs. When the younger sibling was unvaccinated, there was a 69.8% (95% CI 64.9–74.6) chance that the older sibling would also be unvaccinated. In contrast, when the younger sibling had been vaccinated, only 8.3% (95% CI 7.0–9.6) of the older siblings were in the zero-dose group. Discordant sibling pairs were particularly frequent among poor and rural families (Supplemental Table S9). Sensibility analysis using the subgroups of 12–23 months and 24–35 months showed consistent results (data not presented), as well as the analysis of 40 African countries (Supplemental Table S10).

## Discussion

Identifying factors that influence immunization coverage is essential to provide direction to policymaking and improve vaccination coverage. By analysing 85 countries from all continents, our study includes by far the largest set of analyses on the potential role of birth order and number of siblings as drivers of immunization status.

Our findings show that both birth order and the number of siblings were associated with zero-dose prevalence in the ecological and individual level analyses. After adjustment for sociodemographic characteristics in the individual-level analyses, zero-dose prevalence was about 40% higher in children with four or more siblings compared to single children, and 33% higher among those with a birth order of five or more compared to first-born children. Zero-dose prevalence was higher in households with more children regardless of the index child's birth order, but the reverse was not observed given that when the number of siblings was held constant, there was no clear pattern in zero-dose prevalence by birth order of the index child. These findings are supported by the multivariate analyses including both exposure variables, in which the dose–response association with the number of siblings persisted, but in which later birth order was no longer associated with greater zero-dose prevalence after adjustment for the number of siblings. If anything, within families with a given number of siblings, the adjusted models suggest later birth order is associated with lower zero-dose prevalence. Our within-family analyses confirmed that zero-dose children tended to be clustered in the same families.

The composition of a family can influence how caregiving responsibilities are structured within a household. Previous analyses from several countries showed that birth order was associated with immunization coverage indicators that included zero-dose prevalence, full vaccination coverage or missed opportunities for immunization.^22^^,^^36^, ^37^, ^38^, ^39^ Aligned with the present findings, earlier studies revealed that first-born children tended to have better immunization indicators than children with later birth orders. With one exception, however,^22^ these studies did not include adjustment for the number of siblings.

We identified 31 studies in the existing literature addressing this topic in LMIC contexts. Out of these, 29 reported on birth order, of which only five^40^, ^41^, ^42^, ^43^, ^44^ failed to show inverse associations with immunization coverage indicators. Six studies were identified that investigated how the number of siblings (or family size) affects vaccination status, of which five reported inverse associations.^22^^,^^24^^,^^25^^,^^45^, ^46^, ^47^ Only three studies included simultaneous adjustment for birth order and number of siblings^22^^,^^25^ or family size^24^ with two^22^^,^^24^ showing that associations with birth order remained after adjustment for the number of siblings, and vice-versa.

Two studies were identified that covered more than one country. The first reported on missed opportunities for vaccination in children from 35 sub-Saharan Africa countries with data up to 2016.^22^ Analyses were restricted to children who contacted health services to receive preventive or curative interventions. The authors reported a 16% increase (95% CI 9–24%) in the odds of having missed an opportunity for vaccination among children with birth order of four or later, compared to all other children; the analyses were adjusted for several covariates including the number of siblings. Missed opportunities were also associated with the number of children in the family, with an increased odds of 4% (95% CI 1–5%) per sibling, also in the adjusted model; this is equivalent to an increase of 17% for four additional siblings. This earlier study differs from the present analyses as the outcome was missed opportunities for vaccination among children who had used health services, rather than zero-dose prevalence among all children sampled. When we restricted our analyses to sub-Saharan Africa countries, our results remained virtually unchanged (Supplemental Table S10). The second multicountry study investigated differences in immunization among children in urban and rural areas of 23 Sub-Saharan African countries, showing that children with birth orders of six or later exhibited a lower risk of complete immunization compared to first-born children, in both urban and rural areas.^23^ This study did not report on the number of siblings.

The associations of health service coverage with birth order and number of siblings have been attributed to multiple factors. Family size has been related to the dispersion of available resources and to increased domestic workload. In larger families, parents may face greater challenges in organizing and ensuring the quality of childcare, which can impact the level of healthcare investments and access to services.^37^^,^^39^^,^^48^ Regarding birth order, it has been postulated that some parents might draw from their prior experiences with their oldest child, including adverse reactions or negative experiences with health services. Such negative experiences may lead parents to avoid vaccinating their younger children.^22^^,^^24^^,^^38^^,^^48^^,^^49^ Although such explanations are provided in the literature, these are contradicted by our finding that—regardless of birth order—children from larger families tend to show higher zero-dose prevalence. In other words, first-born children who will later have several younger siblings are already less likely to receive any vaccines even before their siblings are born. Our results thus indicate that there may be structural factors—such as poverty, low parental education and place of residence—which drive both family size and low vaccination coverage. Such structural factors account for a substantial proportion of the associations with birth order and number of siblings observed in the unadjusted analyses. Regardless of the causal pathways, the present results suggest that targeting immunization efforts at children from large families will likely help reduce zero-dose prevalence. This is also relevant for designing catch-up strategies to vaccinate older children, which has gained attention following disruptions to immunisation services during the COVID-19 pandemic, for example through “The Big Catch-up”.^11^ Our findings of a high degree of clustering of zero-dose children within households suggest that if immunization programs reach a zero-dose child, it is likely their siblings are also zero-dose.

In our pooled analyses, there was no indication that the child's sex could potentially modify the association between birth order and zero-dose prevalence. However, such effect modification may be present in specific country contexts. An earlier study from India found that the highest increase in full immunization coverage between 1992 and 2006 was observed among boys with two or more surviving older sisters, whereas the smallest increase was observed among girls with older surviving brothers. These results suggest the existence of sex bias, reflected by greater investments in male than in female children aimed at ensuring that couples have at least one male descendant. Cultural contexts, policy-level initiatives, and systematic limitations within healthcare systems across various countries may contribute to the lack of accessibility to immunisation for girls.^14^ While such type of effect modification may be present in some countries but not in others, sex disparities in child vaccination should continue to be closely monitored.

Our study has limitations. Although 85 countries were included, the representation of upper-middle-income countries was limited to 55% of all world countries in this group, as such countries—where zero-dose prevalence is low - tend to rely on health information systems rather than surveys to monitor vaccine coverage.^7^ Information on vaccination status was obtained by maternal recall when home-based records were not available; this comprised approximately one fourth of the sample, but it is reassuring that the proportion of information based on recall did not vary by birth order or by the number of siblings.

The 2.9% of children with missing values on immunization were classified as unvaccinated as per international recommendations.^30^ This is unlikely to have biased the results given that missing values did not vary substantially according to birth order or number of siblings. Missing variables for explanatory or confounding variables were also uncommon, affecting 1.8% or less of the study sample.

An additional limitation is that the analyses include surveys over a 12-year period (2010–2021), but it is unlikely that the associations reported here would change markedly over time; furthermore, three quarters of the children were studied in a four-year period (2016–2019). Only five countries (4.4% of the sample of children) held surveys in 2020–2021 and therefore we were unable to explore whether the pandemic might have affected the present associations.

Lastly, in light of important collinearity between birth order and the number of siblings as shown by the high value of the variance inflation factor, statistical models when both variables are included must be interpreted with due caution. Nevertheless, our double stratification analyses (Fig. 1) show that zero-dose prevalence among children with the same birth order tends to be higher among those with more siblings, whereas there seems to be no increase in prevalence with birth order when the number of siblings is held constant. This is consistent with the results from Tables 2 and 3, suggesting that our findings are robust in spite of the presence of collinearity.

Our research provides the largest available set of analyses examining how vaccine coverage varies according to birth order and the number of children. We focus on zero-dose children, as the reduction in their numbers is crucial to achieving global immunization goals.^2^ In contrast to prior studies that failed to account for the number of siblings, our analyses suggest that birth order seems to play a less critical role in identifying zero-dose children than family size. The number of children within a family may operate as an autonomous risk factor for the prevalence of zero-dose cases, possibly by limiting the amount of time parents have available to seek health services for each child. It may also be that large families tend to present other inherent characteristics that also limit access to care. Whatever the explanation, this finding is reinforced by the intra-family clustering of such children.

Our results suggest that nations with high zero-dose prevalence should consider targeting families with many children. Such large families are easy to identify within any community and reaching them and assessing the vaccination status of all their children—regardless of their age–is essential for ensuring that all children receive the crucial benefits of immunization. Lastly, we recommend that when unvaccinated children are identified by health workers, their siblings should be traced in order to assess and complement their immunizations if necessary.

## Contributors

FSC contributed on the methodology, did the formal analyses, interpreted the results, and contributed to writing the original draft and editing; LANS, BOC-P, TMS, and LZF contributed on the methodology, did the formal analyses, and interpreted the results; TM and DRH conceptualized the study, interpreted the results, contributed to writing and review of the manuscript; AJDB conceptualized the study, contributed on the methodology, supervised the analyses, and critically reviewed the manuscript; CGV conceptualized the study, contributed on the methodology, supervised the analyses, and wrote the manuscript. The data were accessed and verified by FSC, LANS, BOC-P, and TMS. All authors read and approved the final version of the manuscript.

## Data sharing statement

Individual level data used in these analyses are publicly available at the DHS (dhsprogram.com) and the MICS (mics.unicef.org) websites.

## Declaration of interests

TM and DHR are employed by Gavi, the Vaccine Alliance, funder of this research. All other authors declare no competing interests.
