# Supplementary material for: Child immunization status according to number of siblings and birth order in 85 low- and middle-income countries: a cross-sectional study
Source: eClinicalMedicine. 2024 Mar 16;71:102547. doi: 10.1016/j.eclinm.2024.102547 (PMC10958219; doi:10.1016/j.eclinm.2024.102547)

**Table of contents.** Supplementary materials

| **Material** | **Description** | **Page** |
| --- | --- | --- |
| **Supplemental Table 1** | List of countries, surveys, dates, region of the world and respective number of children aged 12-35 months evaluated, and proportion of zero-dose children. | 3 |
| **Supplemental Table 2** | Distribution of surveys according to year of data collection. | 6 |
| **Supplemental Table 3** | Percentages of children with missing information on immunizations according to birth order, number of siblings and household wealth quintiles, by country. | 7 |
| **Supplemental Table 4** | Sample description according to demographic, socioeconomic characteristics, and child immunization (n=375,548) | 10 |
| **Supplemental Table 5** | Proportion of children with immunization information based upon maternal recall, by birth order and number of siblings. | 12 |
| **Supplemental Table 6** | Pearson correlation coefficients between number of siblings and birth order. | 13 |
| **Supplemental Table 7** | Zero-dose prevalence by birth order and sex of the child (p-value for interaction = 0.852). | 15 |
| **Supplemental Table 8** | Crude zero-dose prevalence ratios (PR) according to birth order and number of siblings stratified by survey source. | 16 |
| **Supplemental Table 9** | Characterization of discordant pairs (younger sibling *versus* older sibling) according to number of siblings and birth order. | 17 |
| **Supplemental Table 10** | Zero-dose prevalence ratios (PR) according to birth order among children from sub-Saharan Africa  (n= 149,046) | 18 |
| **Supplemental Table 11** | Zero-dose prevalence ratios (PR) according to the number of living siblings. | 19 |
| **Supplemental Box 1** | List of studies assessed in the systematic literature review and their main results. | 20 |
| **Supplemental Figure 1** | Ecological analyses of average birth order and average number of siblings, with 85 countries as the units of analysis. Pearson’s correlation coefficient = 0.994 (95% CI 0.990 to 0.996; p-value<0.001). | 26 |
| **Supplemental Figure 2** | Ecological analyses of zero-dose prevalence according to national gross domestic product per capita (GDP), with 85 countries as the units of analysis. Pearson’s correlation coefficient = -0.332 (95% CI -0.509 to -0.128; p-value<0.001). | 27 |
| **Supplemental Figure 3** | Ecological analyses of zero-dose prevalence according to national mean number of siblings, with 85 countries as the units of analysis. Pearson’s correlation coefficient = 0.506 (95% CI 0.328 to 0.649; p-value<0.001). | 28 |
| **Supplemental Figure 4** | Ecological analyses of zero-dose prevalence according to national mean number of siblings, with 85 countries as the units of analysis. *Note: The bars represent the prevalence of zero-dose and the whiskers the 95% Confidence Interval.* | 29 |
| **Supplemental Figure 5** | Ecological analyses of zero-dose prevalence according to national mean birth order, with 85 countries as the units of analysis. Pearson’s correlation coefficient = 0.496 (95% CI 0.316 to 0.641; p-value<0.001). | 30 |
| **Supplemental Figure 6** | Ecological analyses of zero-dose prevalence according to national mean birth order, with 85 countries as the units of analysis. *Note: The bars represent the prevalence of zero-dose and the whiskers the 95% Confidence Interval.* | 31 |

**Supplemental Table 1.** List of countries, surveys, dates, region of the world and respective number of children aged 12-35 months evaluated, and proportion of zero-dose children (95% confidence intervals).

| **Country** | **Year** | **Survey** | **Number of children**  **(unweighted)** | **Zero-dose prevalence***  **(Weighted¹)** | | |
| --- | --- | --- | --- | --- | --- | --- |
|  |  |  |  | **%** | **95% CI** | |
| **East Asia & the Pacific** |  |  | **30192** | **10.5** | **9.7;** | **11.4** |
| Tuvalu | 2019 | MICS | 213 | 2.9 | 1.3; | 6.2 |
| Mongolia | 2018 | MICS | 2301 | 3.8 | 2.8; | 5.2 |
| Fiji | 2021 | MICS | 827 | 4.0 | 2.8; | 5.7 |
| Vietnam | 2020 | MICS | 1687 | 4.4 | 3.2; | 6.0 |
| Tonga | 2019 | MICS | 527 | 5.3 | 3.3; | 8.5 |
| Cambodia | 2014 | DHS | 2827 | 5.4 | 4.3; | 6.8 |
| Indonesia | 2017 | DHS | 6942 | 10.6 | 9.5; | 11.8 |
| Myanmar | 2015 | DHS | 1815 | 13.0 | 10.5; | 16.1 |
| Timor Leste | 2016 | DHS | 2765 | 23.0 | 20.5; | 25.7 |
| Samoa | 2019 | MICS | 1119 | 25.8 | 22.3; | 29.6 |
| Lao | 2017 | MICS | 4598 | 29.7 | 27.6; | 32.0 |
| Papua New Guinea | 2016 | DHS | 3693 | 36.1 | 32.9; | 39.3 |
| Kiribati | 2018 | MICS | 878 | 42.7 | 38.2; | 47.2 |
|  |  |  |  |  |  |  |
| **Eastern & Southern Africa** |  |  | **63694** | **15.3** | **14.0;** | **16.7** |
| Rwanda | 2019 | DHS | 3159 | 0.5 | 0.3; | 0.8 |
| Burundi | 2016 | DHS | 4980 | 1.0 | 0.7; | 1.3 |
| Zambia | 2018 | DHS | 3813 | 2.3 | 1.7; | 3.1 |
| Kenya | 2014 | DHS | 8070 | 2.6 | 2.2; | 3.1 |
| Tanzania | 2015 | DHS | 4034 | 3.5 | 2.6; | 4.7 |
| Eswatini | 2014 | MICS | 1118 | 4.5 | 3.3; | 6.0 |
| Zimbabwe | 2019 | MICS | 2401 | 5.5 | 4.2; | 7.3 |
| Uganda | 2016 | DHS | 5838 | 6.1 | 5.3; | 7.0 |
| Namibia | 2013 | DHS | 1973 | 6.8 | 5.5; | 8.5 |
| Malawi | 2019 | MICS | 6404 | 6.9 | 6.0; | 7.9 |
| Lesotho | 2018 | MICS | 1373 | 9.6 | 7.8; | 11.7 |
| South Africa | 2016 | DHS | 1346 | 10.6 | 8.5; | 13.3 |
| Mozambique | 2015 | DHS | 2034 | 11.7 | 9.2; | 14.8 |
| Comoros | 2012 | DHS | 1210 | 20.4 | 16.9; | 24.3 |
| Madagascar | 2021 | DHS | 4662 | 22.5 | 20.5; | 24.7 |
| Ethiopia | 2019 | DHS | 2101 | 29.7 | 25.0; | 34.8 |
| Angola | 2015 | DHS | 5524 | 32.2 | 29.9; | 34.7 |
| South Sudan** | 2010 | MICS | 3654 | 72.7 | 69.6; | 75.5 |
|  |  |  |  |  |  |  |
| **Eastern Europe & Central Asia** |  |  | **8717** | **5.0** | **4.0;** | **6.1** |
| Turkmenistan | 2015 | MICS | 1523 | 0.6 | 0.3; | 1.1 |
| Armenia | 2015 | DHS | 677 | 2.5 | 1.5; | 4.3 |
| North_Macedonia | 2018 | MICS | 598 | 3.0 | 1.5; | 6.0 |
| Kosovo | 2019 | MICS | 596 | 3.9 | 2.5; | 5.9 |
| Turkey | 2013 | DHS | 1418 | 4.8 | 3.4; | 6.6 |
| Kyrgyzstan | 2018 | MICS | 1361 | 7.1 | 5.5; | 9.2 |
| Tajikistan | 2017 | DHS | 2544 | 7.9 | 6.5; | 9.5 |
|  |  |  |  |  |  |  |
| **Latin America & Caribbean** |  |  | **38396** | **6.1** | **5.7** | **6.5** |
| El Salvador | 2014 | MICS | 3014 | 1.1 | 0.7; | 1.8 |
| Guatemala | 2014 | DHS | 4856 | 2.0 | 1.6; | 2.6 |
| Colombia | 2010 | DHS | 7003 | 3.0 | 2.5; | 3.7 |
| Honduras | 2019 | MICS | 3274 | 3.8 | 3.0; | 4.7 |
| Paraguay | 2016 | MICS | 1939 | 6.2 | 5.0; | 7.7 |
| Peru | 2021 | DHS | 8755 | 7.3 | 6.5; | 8.2 |
| Belize | 2015 | MICS | 1029 | 8.1 | 5.6; | 11.5 |
| Dominican Republic | 2019 | MICS | 3340 | 10.1 | 8.8; | 11.6 |
| Guyana | 2019 | MICS | 1055 | 12.5 | 9.8; | 15.8 |
| Haiti | 2016 | DHS | 2432 | 18.6 | 16.1; | 21.4 |
| Suriname | 2018 | MICS | 1699 | 22.8 | 19.6; | 26.3 |
|  |  |  |  |  |  |  |
| **Middle East & North Africa** |  |  | **38042** | **9.3** | **8.6;** | **9.8** |
| Egypt | 2014 | DHS | 6409 | 0.8 | 0.5; | 1.2 |
| State of Palestine | 2019 | MICS | 2625 | 4.6 | 3.8; | 5.7 |
| Algeria | 2018 | MICS | 5881 | 5.2 | 4.4; | 6.0 |
| Tunisia | 2018 | MICS | 1327 | 5.8 | 4.6; | 7.4 |
| Jordan | 2017 | DHS | 4017 | 8.0 | 6.4; | 9.9 |
| Iraq | 2018 | MICS | 6347 | 14.3 | 12.8; | 16.0 |
| Sudan | 2014 | MICS | 5288 | 17.2 | 14.8; | 19.9 |
| Yemen | 2013 | DHS | 6148 | 24.9 | 22.9; | 27.0 |
|  |  |  |  |  |  |  |
| **South Asia** |  |  | **111155** | **8.6** | **8.1;** | **9.2** |
| Bangladesh | 2017 | DHS | 3321 | 1.6 | 1.0; | 2.4 |
| India | 2019 | DHS | 87622 | 6.7 | 6.4; | 7.0 |
| Maldives | 2016 | DHS | 1189 | 9.2 | 7.1; | 12.0 |
| Nepal | 2019 | MICS | 2619 | 13.8 | 11.9; | 15.9 |
| Pakistan | 2017 | DHS | 3867 | 15.3 | 12.7; | 18.4 |
| Afghanistan | 2015 | DHS | 12537 | 31.7 | 28.2; | 35.4 |
|  |  |  |  |  |  |  |
| **West & Central Africa** |  |  | **87323** | **27.7** | **26.4;** | **29.0** |
| Gambia | 2019 | DHS | 3086 | 2.2 | 1.6; | 3.0 |
| Senegal | 2019 | DHS | 2398 | 4.2 | 3.1; | 5.8 |
| São Tome and Principe | 2019 | MICS | 754 | 4.3 | 2.9; | 6.2 |
| Sierra Leone | 2019 | DHS | 3590 | 5.8 | 4.9; | 7.0 |
| Burkina Faso | 2010 | DHS | 5467 | 6.0 | 4.9; | 7.2 |
| Ghana | 2017 | MICS | 3416 | 6.2 | 5.1; | 7.6 |
| Guinea Bissau | 2018 | MICS | 2881 | 8.5 | 7.2; | 10.1 |
| Liberia | 2019 | DHS | 2036 | 9.7 | 7.7; | 12.0 |
| Togo | 2017 | MICS | 1961 | 10.3 | 8.5; | 12.5 |
| Mauritania | 2019 | DHS | 4347 | 12.8 | 11.0; | 14.9 |
| Gabon | 2012 | DHS | 2345 | 15.0 | 12.1; | 18.5 |
| Congo Brazzaville | 2014 | MICS | 3603 | 15.3 | 13.6; | 17.3 |
| Benin | 2017 | DHS | 4865 | 16.7 | 14.9; | 18.5 |
| Cameroon | 2018 | DHS | 3577 | 17.0 | 14.8; | 19.5 |
| Mali | 2018 | DHS | 3675 | 18.2 | 15.7; | 20.9 |
| Cote dIvoire | 2016 | MICS | 3499 | 23.3 | 21.3; | 25.4 |
| Niger | 2021 | DHS | 3235 | 23.6 | 19.7; | 27.8 |
| Congo Democratic Republic | 2017 | MICS | 8398 | 33.6 | 29.9; | 37.5 |
| Nigeria | 2018 | DHS | 11893 | 36.4 | 34.6; | 38.2 |
| Guinea | 2018 | DHS | 2677 | 36.9 | 33.7; | 40.2 |
| Chad | 2014 | DHS | 6200 | 43.1 | 40.6; | 45.6 |
| CAR | 2018 | MICS | 3420 | 45.1 | 42.3; | 48.0 |
| **All children** |  |  | **375,548** | **13.7** | **13.3;** | **14.1** |

¹ The estimate for each country was weighted by surveys’ sampling weights and pooled estimates were weighted by the national populations of children aged 12-35 months.

*Ordered from lowest to highest

**Proportion calculated for South Sudan based on children aged 12-23 months because the survey did not collect vaccination data to children aged 24-35 months

**Supplemental Table 2.** Distribution of surveys according to year of data collection and source.

|  | **Surveys** | | | **Children aged 12-35 months**  **(n=375,548)** | | |
| --- | --- | --- | --- | --- | --- | --- |
| **Period** | **Total**  **N (%)** | **DHS**  **N (%)** | **MICS**  **N (%)** | **Total**  **(% weighted)** | **DHS**  **(% weighted)** | **MICS**  **(% weighted)** |
| **2010-2015** | 25 (29.4) | 18 (21.2) | 7 (8.2) | 20.0 | 17.7 | 2.4 |
| **2016-2019** | 55 (64.7) | 26 (30.6) | 29 (34.1) | 75.6 | 63.7 | 11.9 |
| **2020-2021** | 5 (5.9) | 3 (3.5) | 2 (2.4) | 4.4 | 2.6 | 1.8 |
| **Total** | 85 (100.0) | 47 (55.2) | 38 (44.8) | 100.0 | 83.9 | 16.1 |

DHS - Demographic and Health Surveys; MICS - Multiple Indicator Cluster Surveys

**Supplemental Table 3.** Percentages of children with missing information on immunizations according to birth order, number of siblings and household wealth quintiles, by country.

|  | **Proportions of children with missing information on immunizations** | | | | | | | | | | | | | | | |
| --- | --- | --- | --- | --- | --- | --- | --- | --- | --- | --- | --- | --- | --- | --- | --- | --- |
| **Country** | **All children** | **Number of siblings (%)** | | | | | **Birth Order (%)** | | | | | **Wealth Quintiles (%)** | | | | |
|  | **%** | *0* | *1* | *2* | *3* | *4+* | *1* | *2* | *3* | *4* | *5+* | *1* | *2* | *3* | *4* | *5* |
| **East Asia & the Pacific** |  |  |  |  |  |  |  |  |  |  |  |  |  |  |  |  |
| Vietnam | 0.0 | 0.0 | 0.0 | 0.0 | 0.0 | 0.0 | 0.0 | 0.0 | 0.0 | 0.0 | 0.0 | 0.0 | 0.0 | 0.0 | 0.0 | 0.0 |
| Cambodia | 0.1 | 0.1 | 0.1 | 0.0 | 0.6 | 0.0 | 0.1 | 0.1 | 0.0 | 0.7 | 0.0 | 0.2 | 0.4 | 0.0 | 0.0 | 0.0 |
| Timor Leste | 0.3 | 0.0 | 0.8 | 0.5 | 0.3 | 0.0 | 0.0 | 1.1 | 0.3 | 0.4 | 0.0 | 0.3 | 0.3 | 0.5 | 0.2 | 0.2 |
| Indonesia | 0.5 | 0.6 | 0.5 | 0.4 | 0.4 | 0.8 | 0.5 | 0.5 | 0.4 | 0.4 | 0.8 | 0.4 | 0.6 | 0.5 | 0.4 | 0.5 |
| Myanmar | 0.5 | 0.5 | 0.9 | 0.4 | 0.0 | 0.2 | 0.5 | 0.9 | 0.4 | 0.0 | 0.2 | 0.7 | 0.0 | 0.8 | 1.1 | 0.0 |
| Tonga | 0.5 | 0.0 | 0.0 | 0.0 | 0.0 | 2.5 | 0.0 | 0.0 | 0.0 | 0.0 | 2.8 | 0.0 | 0.0 | 2.9 | 0.0 | 0.0 |
| Mongolia | 2.0 | 1.6 | 2.2 | 2.2 | 0.9 | 1.1 | 1.8 | 1.8 | 2.2 | 0.8 | 1.6 | 2.0 | 1.6 | 2.3 | 2.7 | 1.4 |
| Papua New Guinea | 2.6 | 2.3 | 2.6 | 2.6 | 4.5 | 1.9 | 2.6 | 2.1 | 2.7 | 5.5 | 1.5 | 4.2 | 1.1 | 3.7 | 1.8 | 2.3 |
| Tuvalu | 2.9 | 6.3 | 0.0 | 0.0 | 5.4 | 0.0 | 4.6 | 2.8 | 2.7 | 0.0 | 0.0 | 6.8 | 2.3 | 0.0 | 0.0 | 5.8 |
| Fiji | 3.5 | 4.4 | 2.5 | 3.0 | 0.8 | 5.2 | 4.4 | 2.5 | 2.6 | 0.0 | 6.7 | 3.5 | 4.1 | 4.4 | 3.8 | 0.9 |
| Samoa | 20.3 | 15.5 | 23.0 | 15.2 | 14.4 | 22.7 | 18.8 | 21.6 | 12.5 | 19.5 | 19.8 | 23.1 | 25.5 | 17.6 | 18.1 | 14.7 |
| Lao | 25.2 | 18.6 | 24.7 | 23.6 | 27.3 | 37.8 | 20.0 | 24.3 | 23.5 | 28.9 | 38.3 | 39.8 | 28.1 | 19.1 | 17.0 | 15.3 |
| Kiribati | 42.2 | 39.4 | 38.9 | 45.0 | 39.3 | 46.2 | 35.8 | 42.2 | 42.7 | 41.7 | 43.8 | 43.6 | 35.2 | 43.5 | 50.0 | 39.0 |
|  |  |  |  |  |  |  |  |  |  |  |  |  |  |  |  |  |
| **Eastern & Southern Africa** |  |  |  |  |  |  |  |  |  |  |  |  |  |  |  |  |
| Rwanda | 0.0 | 0.0 | 0.0 | 0.0 | 0.0 | 0.0 | 0.0 | 0.0 | 0.0 | 0.0 | 0.0 | 0.0 | 0.0 | 0.0 | 0.0 | 0.0 |
| Burundi | 0.0 | 0.1 | 0.0 | 0.0 | 0.0 | 0.0 | 0.1 | 0.0 | 0.0 | 0.0 | 0.0 | 0.0 | 0.0 | 0.0 | 0.0 | 0.1 |
| Tanzania | 0.1 | 0.2 | 0.0 | 0.0 | 0.1 | 0.0 | 0.2 | 0.0 | 0.0 | 0.1 | 0.0 | 0.1 | 0.0 | 0.0 | 0.3 | 0.0 |
| Kenya | 0.2 | 0.1 | 0.4 | 0.0 | 0.2 | 0.1 | 0.1 | 0.4 | 0.0 | 0.3 | 0.0 | 0.1 | 0.0 | 0.1 | 0.4 | 0.3 |
| Madagascar | 0.2 | 0.5 | 0.0 | 0.0 | 0.0 | 0.4 | 0.4 | 0.0 | 0.0 | 0.0 | 0.4 | 0.4 | 0.1 | 0.2 | 0.3 | 0.0 |
| Zambia | 0.4 | 0.7 | 0.0 | 0.0 | 0.2 | 0.8 | 0.7 | 0.0 | 0.1 | 0.3 | 0.7 | 0.1 | 0.1 | 0.2 | 1.0 | 0.9 |
| Uganda | 0.5 | 0.9 | 0.6 | 0.1 | 0.2 | 0.5 | 0.7 | 0.6 | 0.1 | 0.8 | 0.3 | 0.5 | 0.2 | 0.6 | 0.6 | 0.5 |
| Ethiopia | 1.6 | 0.8 | 0.7 | 0.7 | 0.1 | 3.5 | 0.6 | 0.8 | 0.8 | 0.0 | 3.6 | 1.6 | 2.8 | 1.3 | 1.2 | 0.9 |
| Namibia | 1.7 | 1.8 | 2.2 | 1.9 | 0.6 | 0.9 | 1.6 | 2.3 | 2.0 | 1.6 | 0.3 | 0.3 | 1.1 | 1.0 | 2.9 | 3.3 |
| Comoros | 1.8 | 1.4 | 0.7 | 4.1 | 1.0 | 1.9 | 1.5 | 3.2 | 1.0 | 1.3 | 1.7 | 1.8 | 1.6 | 2.9 | 2.1 | 0.2 |
| Angola | 2.0 | 2.1 | 2.2 | 2.1 | 1.9 | 1.8 | 2.5 | 1.7 | 2.4 | 1.4 | 2.0 | 1.0 | 1.9 | 2.0 | 2.2 | 3.6 |
| Mozambique | 3.5 | 2.7 | 5.6 | 3.6 | 2.9 | 3.0 | 3.0 | 5.6 | 3.7 | 2.1 | 3.0 | 5.6 | 3.3 | 1.8 | 4.5 | 1.8 |
| Eswatini | 3.8 | 0.6 | 1.7 | 2.9 | 0.7 | 6.6 | 0.2 | 2.1 | 1.0 | 0.8 | 4.1 | 4.1 | 3.6 | 6.1 | 4.4 | 0.3 |
| South Africa | 4.8 | 4.4 | 4.0 | 5.1 | 9.7 | 2.5 | 5.3 | 3.0 | 4.8 | 10.1 | 2.8 | 3.8 | 7.3 | 4.3 | 3.4 | 4.4 |
| Lesotho | 5.1 | 4.1 | 1.0 | 2.2 | 0.6 | 7.4 | 3.7 | 1.6 | 1.2 | 0.0 | 3.9 | 7.5 | 4.4 | 6.3 | 2.8 | 3.8 |
| Zimbabwe | 5.4 | 1.8 | 2.9 | 2.7 | 5.4 | 14.7 | 2.8 | 2.6 | 3.0 | 5.3 | 14.8 | 9.9 | 6.4 | 4.0 | 2.6 | 2.5 |
| Malawi | 5.9 | 4.7 | 5.3 | 5.0 | 4.1 | 8.5 | 4.7 | 5.2 | 4.9 | 6.7 | 6.4 | 6.4 | 5.9 | 6.8 | 6.5 | 3.5 |
| South Sudan | 70.7 | 61.0 | 67.7 | 70.0 | 75.3 | 73.0 | 60.4 | 70.8 | 68.4 | 78.2 | 70.9 | 84.1 | 78.9 | 72.4 | 70.6 | 49.5 |
|  |  |  |  |  |  |  |  |  |  |  |  |  |  |  |  |  |
| **Eastern Europe & Central Asia** |  |  |  |  |  |  |  |  |  |  |  |  |  |  |  |  |
| Kyrgyzstan | 0.0 | 0.0 | 0.0 | 0.0 | 0.0 | 0.0 | 0.0 | 0.0 | 0.0 | 0.0 | 0.0 | 0.0 | 0.0 | 0.0 | 0.0 | 0.0 |
| Turkmenistan | 0.3 | 0.3 | 0.3 | 0.3 | 0.7 | 0.0 | 0.2 | 0.4 | 0.6 | 0.0 | 0.0 | 0.0 | 0.0 | 0.0 | 0.7 | 1.1 |
| North Macedonia | 0.8 | 0.0 | 1.3 | 0.5 | 0.0 | 0.0 | 0.0 | 1.5 | 0.6 | 0.0 | 0.0 | 0.3 | 0.0 | 0.7 | 2.8 | 0.4 |
| Armenia | 0.9 | 0.8 | 1.4 | 0.0 | 0.0 | 0.0 | 0.9 | 1.4 | 0.0 | 0.0 | 0.0 | 0.3 | 1.3 | 1.0 | 1.4 | 0.7 |
| Tajikistan | 1.2 | 1.2 | 1.4 | 1.3 | 1.0 | 1.1 | 1.2 | 1.5 | 1.1 | 1.0 | 0.9 | 1.4 | 0.8 | 0.3 | 1.8 | 2.2 |
| Kosovo | 1.5 | 0.5 | 3.0 | 0.0 | 1.5 | 2.5 | 1.1 | 2.6 | 0.0 | 1.6 | 3.2 | 1.2 | 1.7 | 1.7 | 1.3 | 1.7 |
| Turkey | 2.1 | 0.9 | 2.0 | 1.1 | 7.2 | 3.1 | 2.0 | 1.1 | 1.9 | 6.5 | 3.3 | 2.9 | 3.7 | 2.3 | 0.4 | 0.2 |
|  |  |  |  |  |  |  |  |  |  |  |  |  |  |  |  |  |
| **Latin America & Caribbean** |  |  |  |  |  |  |  |  |  |  |  |  |  |  |  |  |
| Guatemala | 0.1 | 0.3 | 0.0 | 0.0 | 0.0 | 0.0 | 0.3 | 0.0 | 0.0 | 0.0 | 0.0 | 0.0 | 0.1 | 0.1 | 0.3 | 0.0 |
| Haiti | 0.2 | 0.1 | 0.0 | 0.0 | 0.0 | 0.7 | 0.1 | 0.0 | 0.0 | 0.0 | 0.7 | 0.2 | 0.5 | 0.1 | 0.0 | 0.0 |
| Colombia | 0.3 | 0.2 | 0.3 | 0.2 | 0.9 | 0.7 | 0.3 | 0.1 | 0.7 | 0.4 | 0.4 | 0.5 | 0.3 | 0.4 | 0.0 | 0.1 |
| Peru | 0.4 | 0.5 | 0.3 | 0.2 | 1.2 | 0.5 | 0.5 | 0.2 | 0.5 | 0.8 | 0.5 | 0.3 | 0.6 | 0.4 | 0.4 | 0.3 |
| El Salvador | 0.9 | 0.8 | 0.4 | 0.1 | 1.2 | 2.1 | 0.7 | 0.3 | 0.0 | 1.3 | 2.5 | 0.3 | 1.3 | 0.5 | 2.0 | 0.4 |
| Honduras | 3.4 | 3.7 | 2.1 | 3.2 | 2.5 | 4.1 | 3.5 | 1.9 | 2.8 | 3.1 | 4.5 | 2.2 | 2.9 | 4.6 | 4.2 | 3.2 |
| Paraguay | 6.0 | 3.9 | 5.5 | 6.8 | 5.0 | 7.6 | 3.8 | 5.7 | 7.3 | 5.5 | 4.4 | 7.2 | 5.9 | 5.1 | 6.3 | 5.1 |
| Belize | 7.8 | 5.2 | 5.9 | 9.0 | 8.0 | 13.1 | 5.2 | 5.9 | 8.2 | 6.1 | 14.8 | 7.9 | 4.8 | 7.1 | 8.7 | 12.4 |
| Dominican Republic | 8.3 | 5.8 | 6.3 | 10.4 | 9.3 | 12.7 | 5.8 | 6.6 | 9.2 | 8.4 | 11.7 | 13.7 | 9.1 | 5.1 | 6.5 | 5.2 |
| Guyana | 11.1 | 10.0 | 8.0 | 5.7 | 18.3 | 14.3 | 8.1 | 6.1 | 8.0 | 17.1 | 15.1 | 14.4 | 6.4 | 9.8 | 10.6 | 13.0 |
| Suriname | 16.0 | 14.3 | 12.3 | 11.6 | 15.3 | 13.9 | 11.5 | 12.2 | 13.3 | 11.6 | 13.5 | 21.2 | 11.7 | 13.6 | 15.4 | 15.0 |
|  |  |  |  |  |  |  |  |  |  |  |  |  |  |  |  |  |
| **Middle East & North Africa** |  |  |  |  |  |  |  |  |  |  |  |  |  |  |  |  |
| Egypt | 0.0 | 0.0 | 0.0 | 0.0 | 0.0 | 0.0 | 0.0 | 0.0 | 0.0 | 0.0 | 0.0 | 0.0 | 0.0 | 0.0 | 0.0 | 0.0 |
| Jordan | 0.2 | 0.2 | 0.3 | 0.0 | 0.1 | 0.2 | 0.1 | 0.3 | 0.0 | 0.1 | 0.2 | 0.5 | 0.0 | 0.0 | 0.1 | 0.0 |
| Yemen | 0.9 | 1.0 | 0.8 | 1.0 | 0.5 | 0.9 | 0.7 | 0.9 | 1.0 | 0.7 | 0.9 | 1.2 | 0.8 | 0.7 | 0.7 | 0.8 |
| State of Palestine | 2.2 | 2.2 | 2.3 | 2.3 | 2.1 | 2.1 | 2.6 | 1.9 | 2.3 | 2.4 | 1.7 | 0.5 | 1.6 | 2.6 | 3.2 | 3.5 |
| Tunisia | 3.4 | 3.0 | 3.7 | 2.8 | 4.9 | 3.4 | 3.8 | 3.7 | 2.3 | 4.2 | 3.2 | 2.3 | 2.7 | 4.1 | 4.9 | 3.0 |
| Algeria | 3.6 | 3.2 | 2.8 | 4.4 | 2.9 | 5.3 | 2.8 | 3.0 | 4.3 | 3.8 | 5.2 | 5.6 | 2.2 | 3.4 | 3.5 | 2.9 |
| Iraq | 9.3 | 4.6 | 9.3 | 9.5 | 9.9 | 11.5 | 6.0 | 9.4 | 10.6 | 8.3 | 12.0 | 12.6 | 9.0 | 10.3 | 9.6 | 4.0 |
| Sudan | 17.1 | 14.4 | 15.0 | 16.2 | 17.8 | 18.1 | 13.2 | 17.8 | 16.5 | 16.7 | 18.1 | 34.7 | 22.7 | 10.7 | 8.4 | 5.9 |
|  |  |  |  |  |  |  |  |  |  |  |  |  |  |  |  |  |
| **South Asia** |  |  |  |  |  |  |  |  |  |  |  |  |  |  |  |  |
| Bangladesh | 0.1 | 0.0 | 0.0 | 0.1 | 0.7 | 0.0 | 0.0 | 0.1 | 0.0 | 0.7 | 0.0 | 0.0 | 0.0 | 0.0 | 0.3 | 0.0 |
| India | 0.2 | 0.2 | 0.2 | 0.2 | 0.3 | 0.5 | 0.2 | 0.2 | 0.2 | 0.3 | 0.4 | 0.3 | 0.2 | 0.2 | 0.2 | 0.1 |
| Pakistan | 0.3 | 0.0 | 0.6 | 0.0 | 0.2 | 0.3 | 0.2 | 0.4 | 0.0 | 0.2 | 0.3 | 0.6 | 0.1 | 0.3 | 0.1 | 0.1 |
| Maldives | 1.0 | 0.8 | 0.3 | 2.4 | 1.2 | 0.0 | 0.7 | 1.3 | 1.1 | 1.3 | 0.0 | 2.0 | 0.0 | 1.0 | 0.0 | 2.0 |
| Afghanistan | 2.7 | 1.9 | 3.3 | 2.8 | 2.6 | 2.8 | 1.9 | 3.6 | 3.1 | 2.5 | 2.7 | 1.2 | 2.7 | 3.2 | 4.7 | 1.7 |
| Nepal | 12.8 | 8.6 | 12.5 | 12.1 | 20.6 | 28.3 | 9.6 | 12.0 | 13.0 | 23.1 | 24.1 | 17.5 | 17.2 | 9.9 | 7.7 | 10.1 |
|  |  |  |  |  |  |  |  |  |  |  |  |  |  |  |  |  |
| **West & Central Africa** |  |  |  |  |  |  |  |  |  |  |  |  |  |  |  |  |
| Senegal | 0.0 | 0.0 | 0.0 | 0.0 | 0.0 | 0.0 | 0.0 | 0.0 | 0.0 | 0.0 | 0.0 | 0.0 | 0.0 | 0.0 | 0.0 | 0.0 |
| Burkina Faso | 0.1 | 0.2 | 0.1 | 0.1 | 0.2 | 0.0 | 0.2 | 0.1 | 0.1 | 0.2 | 0.0 | 0.2 | 0.0 | 0.1 | 0.0 | 0.0 |
| Gambia | 0.2 | 0.2 | 0.0 | 0.4 | 0.0 | 0.3 | 0.2 | 0.4 | 0.0 | 0.0 | 0.4 | 0.0 | 0.2 | 0.3 | 0.5 | 0.0 |
| Benin | 0.2 | 0.4 | 0.1 | 0.2 | 0.4 | 0.2 | 0.3 | 0.1 | 0.2 | 0.4 | 0.3 | 0.2 | 0.2 | 0.1 | 0.2 | 0.5 |
| Nigeria | 0.3 | 0.4 | 0.1 | 0.3 | 0.4 | 0.3 | 0.3 | 0.1 | 0.3 | 0.5 | 0.3 | 0.4 | 0.1 | 0.4 | 0.3 | 0.2 |
| Liberia | 0.3 | 0.3 | 1.0 | 0.0 | 0.3 | 0.0 | 0.2 | 1.1 | 0.0 | 0.3 | 0.0 | 0.2 | 0.2 | 1.3 | 0.0 | 0.0 |
| Niger | 0.7 | 1.6 | 0.8 | 0.3 | 0.4 | 0.6 | 1.8 | 0.3 | 0.5 | 0.4 | 0.6 | 0.2 | 0.8 | 0.5 | 0.7 | 1.2 |
| Cameroon | 0.7 | 0.6 | 0.8 | 0.6 | 1.0 | 0.6 | 1.0 | 0.1 | 0.7 | 0.9 | 0.7 | 1.1 | 0.2 | 0.6 | 1.0 | 0.6 |
| Mauritania | 0.9 | 1.1 | 1.2 | 0.8 | 0.3 | 0.8 | 1.0 | 1.3 | 0.6 | 0.2 | 0.8 | 1.0 | 0.2 | 1.4 | 1.5 | 0.1 |
| Mali | 0.9 | 0.0 | 0.9 | 1.0 | 1.6 | 0.9 | 0.2 | 0.9 | 1.1 | 1.6 | 0.8 | 0.8 | 0.9 | 1.0 | 1.2 | 0.3 |
| Guinea | 0.9 | 1.1 | 0.2 | 1.1 | 2.3 | 0.5 | 1.1 | 0.0 | 1.2 | 2.6 | 0.4 | 0.5 | 0.5 | 0.4 | 2.1 | 1.3 |
| Sierra Leone | 0.9 | 1.3 | 1.4 | 0.3 | 0.4 | 1.0 | 1.3 | 1.2 | 0.4 | 0.3 | 1.1 | 0.8 | 1.1 | 1.2 | 1.1 | 0.4 |
| Gabon | 2.0 | 1.0 | 1.3 | 1.0 | 1.5 | 4.7 | 1.1 | 1.4 | 0.8 | 3.4 | 4.2 | 1.0 | 3.7 | 1.9 | 0.2 | 3.9 |
| Chad | 2.1 | 2.4 | 1.7 | 2.3 | 2.2 | 2.0 | 2.4 | 1.9 | 2.3 | 2.3 | 1.9 | 2.0 | 2.8 | 1.6 | 1.4 | 2.7 |
| Sao Tome and Principe | 3.2 | 1.2 | 2.5 | 1.9 | 2.8 | 1.9 | 1.6 | 3.4 | 1.3 | 0.6 | 1.3 | 3.3 | 2.9 | 3.2 | 4.8 | 1.1 |
| Ghana | 6.0 | 3.9 | 4.9 | 6.7 | 4.0 | 5.3 | 3.7 | 5.5 | 6.8 | 3.2 | 3.3 | 7.1 | 5.2 | 7.4 | 5.6 | 4.6 |
| Guinea Bissau | 7.8 | 6.8 | 6.3 | 6.1 | 6.8 | 9.6 | 7.2 | 5.8 | 5.9 | 7.1 | 8.5 | 9.2 | 8.4 | 5.8 | 8.8 | 6.0 |
| Togo | 8.6 | 3.2 | 10.1 | 6.3 | 11.7 | 9.2 | 4.0 | 9.7 | 5.9 | 11.4 | 8.3 | 12.3 | 8.9 | 9.7 | 5.7 | 6.0 |
| Congo Brazzaville | 14.0 | 12.0 | 11.3 | 11.1 | 13.8 | 18.0 | 11.6 | 11.6 | 10.6 | 14.8 | 17.1 | 27.5 | 12.9 | 7.1 | 9.4 | 8.7 |
| Cote d’Ivoire | 15.7 | 11.6 | 17.4 | 13.1 | 13.1 | 18.0 | 11.9 | 15.3 | 14.6 | 12.6 | 16.5 | 23.9 | 15.0 | 15.4 | 12.1 | 6.8 |
| Congo Democratic Republic | 31.8 | 29.6 | 30.7 | 35.0 | 28.8 | 33.1 | 28.7 | 32.5 | 33.8 | 28.4 | 33.1 | 45.1 | 39.5 | 32.1 | 23.6 | 11.8 |
| CAR | 42.1 | 37.9 | 38.9 | 44.5 | 39.7 | 42.7 | 39.7 | 37.4 | 43.2 | 39.8 | 42.7 | 56.1 | 53.2 | 45.2 | 30.9 | 14.7 |
| **All children** | **2.9** | **2.4** | **2.6** | **2.8** | **2.7** | **3.4** | **2.4** | **2.6** | **2.7** | **2.7** | **3.2** | **4.1** | **3.4** | **2.8** | **2.3** | **1.6** |

**Ordered from lowest to highest proportion of missing values for immunizations.*

**Supplemental Table 4.** Sample description according to demographic, socioeconomic characteristics, and child immunization (n=375,548)

|  | **Children aged 12-35 months**  **(n=375,548)** | | |
| --- | --- | --- | --- |
| **Variable^§^** | **n (unweighted)** | **% weighted** | **95% CI** |
| **Area of residence** |  |  |  |
| Urban | 125460 | 34.8 | 34.4; 35.2 |
| Rural | 250088 | 65.2 | 64.7; 65.6 |
| **Family type** |  |  |  |
| Nuclear | 199052 | 52.7 | 52.3; 53.0 |
| Extended | 172388 | 47.3 | 47.0; 47.7 |
| **Wealth quintiles** |  |  |  |
| 1 (poorest) | 97554 | 22.5 | 22.1; 22.9 |
| 2 | 84057 | 21.3 | 20.9; 21.6 |
| 3 | 74284 | 20.2 | 19.8; 20.5 |
| 4 | 65558 | 18.9 | 18.5; 19.2 |
| 5 (richest) | 54095 | 17.2 | 16.8; 17.6 |
| **Maternal age** |  |  |  |
| 15-17 years | 17726 | 4.4 | 4.3; 4.5 |
| 18-19 years | 32050 | 8.5 | 8.4; 8.7 |
| 20-34 years | 275231 | 76.2 | 76.0; 76.5 |
| 35-49 years | 44495 | 10.8 | 10.6; 11.0 |
| **Maternal education** |  |  |  |
| None | 102393 | 26.1 | 25.6; 26.5 |
| Primary | 94,620 | 26.5 | 23.2; 23.8 |
| Secondary + | 178469 | 50.4 | 50.0; 50.9 |
| **Child’s sex** |  |  |  |
| Female | 183643 | 48.9 | 48.5; 49.2 |
| Male | 191905 | 51.1 | 50.8; 51.4 |
| **Birth Order** |  |  |  |
| 1^st^ | 105715 | 29.9 | 29.6; 30.2 |
| 2^nd^ | 92848 | 26.7 | 26.5; 27.0 |
| 3^rd^ | 61147 | 16.2 | 16.0; 16.4 |
| 4^th^ | 39565 | 10.1 | 9.9; 10.3 |
| >=5^th^ | 69671 | 17.0 | 16.7; 17.3 |
| **Number of siblings** |  |  |  |
| None | 84094 | 23.7 | 23.4; 24.0 |
| 1 | 101318 | 29.2 | 28.9; 29.5 |
| 2 | 66008 | 17.5 | 17.3; 17.8 |
| 3 | 43059 | 10.9 | 10.7; 11.1 |
| >=4 | 76554 | 18.7 | 18.4; 19.0 |
| **Vaccine source (maternal recall)** |  |  |  |
| No | 291547 | 75.3 | 74.9; 75.6 |
| Yes | 84001 | 24.7 | 24.4; 25.1 |

*95% CI: 95% Confidence Interval; ^§^ missing data on maternal age (n=6,046), maternal education (n=66),*

*family size (n=4,108), birth order (n=6,602), number of siblings (n=4,515)*

**Supplemental Table 5**. Proportions of children (95% confidence intervals) with immunization information based upon maternal recall, by birth order and number of siblings.

| **Birth order** |  |
| --- | --- |
|  | **Recall (%)** |
| 1^st^ | 24.4 (23.8;25.1) |
| 2^nd^ | 24.4 (23.0.8;25) |
| 3^rd^ | 25.1 (24.4;25.7) |
| 4^th^ | 25.3 (24.5;26.1) |
| >=5^th^ | 24.7 (24.0;25.4) |
| **Number of siblings** |  |
|  | **Recall (%)** |
| None | 23.7 (23.1;24.3) |
| 1 | 24.4 (23.8;24.9) |
| 2 | 25.4 (24.0.8;26) |
| 3 | 25.6 (24.8;26.4) |
| >=4 | 25.0 (24.3;25.7) |

**Supplemental Table 6.** Pearson correlation coefficients between number of siblings and birth order.

| **Country** | **Pearson coefficient (r)** |
| --- | --- |
| Yemen | 0.8379 |
| Chad | 0.8449 |
| Samoa | 0.8460 |
| Sudan | 0.8480 |
| Afghanistan | 0.8513 |
| Niger | 0.8519 |
| Nigeria | 0.8568 |
| Comoros | 0.8594 |
| Congo Democratic Republic | 0.8594 |
| Uganda | 0.8609 |
| Gabon | 0.8615 |
| Mali | 0.8629 |
| Mauritania | 0.8630 |
| Pakistan | 0.8637 |
| Tanzania | 0.8659 |
| Angola | 0.8696 |
| CAR | 0.8746 |
| State of Palestine | 0.8776 |
| South Sudan | 0.8777 |
| Burundi | 0.8782 |
| Cameroon | 0.8786 |
| Kenya | 0.8803 |
| Belize | 0.8811 |
| Congo Brazzaville | 0.8822 |
| Ethiopia | 0.8823 |
| Tuvalu | 0.8826 |
| Gambia | 0.8837 |
| Timor Leste | 0.8843 |
| Madagascar | 0.8851 |
| Burkina Faso | 0.8852 |
| Togo | 0.8857 |
| Mozambique | 0.8860 |
| Cote d’Ivoire | 0.8861 |
| Guatemala | 0.8864 |
| Benin | 0.8875 |
| Iraq | 0.8878 |
| Tonga | 0.8888 |
| Senegal | 0.8899 |
| Lao | 0.8924 |
| Zambia | 0.8925 |
| Haiti | 0.8935 |
| Guinea | 0.8936 |
| Turkey | 0.8946 |
| Liberia | 0.8948 |
| Suriname | 0.8949 |
| Papua New Guinea | 0.8958 |
| Kosovo | 0.8978 |
| Ghana | 0.8998 |
| Jordan | 0.9000 |
| Eswatini | 0.9027 |
| Kiribati | 0.9040 |
| Sierra Leone | 0.9065 |
| Honduras | 0.9078 |
| Myanmar | 0.9085 |
| Armenia | 0.9086 |
| Colombia | 0.9101 |
| Rwanda | 0.9116 |
| Turkmenistan | 0.9117 |
| Guyana | 0.9118 |
| Tunisia | 0.9121 |
| Fiji | 0.9124 |
| Guinea Bissau | 0.9160 |
| Egypt | 0.9165 |
| India | 0.9171 |
| Namibia | 0.9185 |
| Dominican Republic | 0.9201 |
| Paraguay | 0.9204 |
| Kyrgyzstan | 0.9208 |
| Sao Tome and Principe | 0.9221 |
| Algeria | 0.9225 |
| Malawi | 0.9229 |
| El Salvador | 0.9231 |
| Zimbabwe | 0.9250 |
| Tajikistan | 0.9256 |
| Cambodia | 0.9262 |
| Indonesia | 0.9299 |
| South Africa | 0.9359 |
| Vietnam | 0.9366 |
| Peru | 0.9368 |
| Lesotho | 0.9389 |
| Maldives | 0.9404 |
| Mongolia | 0.9437 |
| Nepal | 0.9446 |
| North Macedonia | 0.9450 |
| Bangladesh | 0.9502 |

**Supplemental Table 7**. Zero-dose prevalence and 95% confidence intervals by birth order and sex of the child (p-value for interaction = 0.852).

| **Birth order** | **Boys** | **Girls** |
| --- | --- | --- |
|  | **% (95% CI)** | **% (95% CI)** |
| 1^st^ | 10.7 (10.1;11.5) | 11.3 (10.6;12.0) |
| 2^nd^ | 11.9 (11.3;12.0) | 13.0 (12.2;13.7) |
| 3^rd^ | 13.5 (12.7;14.3) | 13.9 (13.1;14.7) |
| 4^th^ | 14.0 (12.9;15.0) | 14.6 (13.7;15.5) |
| >=5^th^ | 16.7 (15.9;17.6) | 17.4 (16.6;18.2) |
| p-value* | <0.001 | <0.001 |

**P-value for heterogeneity in zero-dose prevalence by birth order, for each sex.*

**Supplemental Table 8**. Crude zero-dose prevalence ratios (PR) and 95% confidence intervals (CI) according to birth order and number of siblings stratified by survey source.

| **Birth order** | **MICS** | **DHS** |
| --- | --- | --- |
|  | **PR (95% CI)** | **PR (95% CI)** |
| 1^st^ | 1.00 | 1.00 |
| 2^nd^ | 1.22 (1.11;1.33) | 1.11 (1.05;1.18) |
| 3^rd^ | 1.24 (1.13;1.37) | 1.25 (1.17;1.33) |
| 4^th^ | 1.19 (1.07;1.32) | 1.33 (1.24;1.44) |
| >=5^th^ | 1.33 (1.20;1.46) | 1.62 (1.51;1.73) |
| **Number of siblings** | **MICS** | **DHS** |
|  | **PR (95% CI)** | **PR (95% CI)** |
| Zero | 1.00 | 1.00 |
| One | 1.21 (1.09;1.35) | 1.15 (1.08;1.23) |
| Two | 1.28 (1.16;1.41) | 1.30 (1.22;1.39) |
| Three | 1.22 (1.09;1.36) | 1.39 (1.28;1.50) |
| >=Four | 1.38 (1.24;1.53) | 1.71 (1.59;1.84) |

**Supplemental Table 9**. Frequency of discordant pairs (younger sibling *versus* older sibling) according to number of siblings and birth order, with 95% confidence intervals.

|  | **Discordant pairs (%)** | **95% CI** |
| --- | --- | --- |
| **Wealth quintiles** |  |  |
| 1 (poorest) | 14.9 | 11.7;18.2 |
| 2 | 12.1 | 9.6;14.7 |
| 3 | 11.9 | 9.1;14.8 |
| 4 | 10.6 | 7.9;13.4 |
| 5 (richest) | 7.4 | 4.1;10.6 |
| **Area of residence** |  |  |
| Urban | 9.9 | 7.4;12.5 |
| Rural | 12.8 | 11.0;14.5 |

**Supplemental Table 10**. Zero-dose prevalence ratios (PR) and 95% confidence intervals (CI) according to birth order among children in 40 sub-Saharan African countries (n= 149,046)

| **Birth order** | **Model 1** | **Model 2** | **Model 3** |
| --- | --- | --- | --- |
|  | **PR (95% CI)** | **PR (95% CI)** | **PR (95% CI)** |
| 1^st^ | 1.00 | 1.00 | 1.00 |
| 2^nd^ | 1.11 (1.02;1.20) | 1.14 (1.06;1.23) | 1.04 (0.95;1.13) |
| 3^rd^ | 1.15 (1.07;1.24) | 1.16 (1.07;1.27) | 0.97 (0.84;1.12) |
| 4^th^ | 1.17 (1.08;1.28) | 1.12 (1.03;1.23) | 0.89 (0.74;1.07) |
| >=5^th^ | 1.41 (1.31;1.53) | 1.21 (1.11;1.32) | 0.84 (0.68;1.05) |
| p-value | <0.001 | <0.001 | 0.011 |

*Model 1 – crude.*

*Model 2 – adjusted for wealth quintiles, maternal education, maternal age at birth, area of residence, family size, vaccine recall, child’s age, and sex.*

*Model 3 – model 2 plus number of siblings.*

**Supplemental Table 11.** Zero-dose prevalence ratios (PR) and 95% confidence intervals (CI) according to the number of living siblings.

| **Number of siblings** | **Model 1** | **Model 2** | **Model 3** |
| --- | --- | --- | --- |
|  | **PR (95% CI)** | **PR (95% CI)** | **PR (95% CI)** |
| None | 1 | 1 | 1 |
| 1 | 1.13 (1.06;1.20) | 1.15 (1.08;1.22) | 1.13 (1.04;1.22) |
| 2 | 1.27 (1.19;1.35) | 1.25 (1.17;1.34) | 1.23 (1.11;1.36) |
| 3 | 1.42 (1.32;1.53) | 1.31 (1.21;1.41) | 1.33 (1.17;1.50) |
| >=4 | 1.62 (1.51;1.74) | 1.38 (1.27;1.49) | 1.35 (1.19;1.55) |
| p-value | <0.001 | < 0.001 | < 0.001 |

*Model 1 – Adjusted for country using fixed effects.*

*Model 2 – Also adjusted for wealth quintiles, maternal education, maternal age at birth, area of residence, family size, vaccine recall, child’s age, and sex.*

*Model 3 – Model 2 plus birth order.*

***Interpretation****: In the body of the manuscript, the analyses only include surviving siblings. This was an a priori decision given that one does not know whether siblings who died were still alive at the time when the index child should have been immunized. In the table above, only siblings who were alive at the time of the interview are counted. The dose-response trend with zero-dose prevalence remained, but the prevalence ratios were attenuated relative to those presented in the main paper. A possible reason for the attenuation is that children with a dead sibling (who are probably at high risk for zero-dose) were moved to lower categories of number of siblings in the table above. The baseline group (no siblings) now includes high-risk children whose siblings have died, while children with say 4+ siblings belong to families with lower mortality (as they have several surviving siblings). The combination of these two events likely explains the reduced risks observed in the table above compared to the analyses considering surviving siblings.*

**Supplemental Box 1.** List of studies assessed in the systematic literature review and their main results.

| **Authors** | **DOI** | **Country** | **Associated with birth order** | **Associated with number of siblings/ family size** | **Mutual adjustment (birth order and siblings)** | **Outcome** | **Observations** |
| --- | --- | --- | --- | --- | --- | --- | --- |
| Ameyaw EK,  Kareem YO, Ahankaras BO, Seidu A, Yaya S | 10.1136/bmjgh-2020-003773 | 23 sub-Saharan African counties | Associated | Not investigated | Not investigated | Full immunization among children aged 12-23 months | Later birth order was associated with a reduced risk of complete immunization. |
| Uthman OA, Sambala EZ, Adamu AA et al. | 10.1080/21645515.2018.1504524 | 35 sub-Saharan African counties | Associated | Associated | Yes, both associated | Missed opportunities for vaccination among children aged 12-23 months | High birth order and high number of under-five children in the house were associated with a higher risk for missed opportunities for  Vaccination. |
| Hanifi SMA, Ravn H, Aaby P, Bhuiya A | 10.1016/j.vaccine.2018.04.059 | Bangladesh | Associated | Not investigated | Not investigated | Full vaccination among children aged 12-23 months | The ratio Female/Male was 1.01 (0.96-1.06) for first born children, but 0.95 (0.93-0.98) for second or later birth order children (p = 0.04, test for interaction). |
| Hossain MM, Sobhan MA, Rahman A, Flora SS, Irin ZS | 10.1186/s12889-021-11576-0 | Bangladesh | Associated | Not investigated | Not investigated | BCG vaccination among children aged 06-59 months | DPT vaccination coverage was significantly decreased with later birth order. |
| Nda’chi Deffo R, Kamga BF | 10.1186/s12913-020-05745-x | Cameroon | Associated | Not investigated | Not investigated | Full vaccination among children under 5 | The probability for the child to be fully immunized decreases with an increase in birth order. |
| Bago BJ,  Terefe W,  Mirutse G | (..) | Ethiopia | Associated | Not investigated | Not investigated | Defauting of immunization among children aged 12-59 months | Later birth order is associated with greater protection against defaulting on immunization |
| Dheresa M, Dessie, Y,  Negash B, Balis B, Getachew T, Ayana GM et al. | 10.2147/JMDH.S325705 | Ethiopia | Associated | Not investigated | Not investigated | Being partially vaccinated among children aged 12-24 months | As the birth  order of the child increased by one, the odds of partially  vaccinated was increased by 4%. |
| Geweniger A, Abbas KM | 10.1016/j.vaccine.2020.03.040 | Ethiopia | Not associated | Not investigated | Not investigated | Full vaccination coverage among children aged 12-23 months | The Odds of full vaccination was 1.7 times higher among first to 3rd born than 4th born or later (reference). |
| Hailu, C.  Fisseha, G.  Gebreyesus, A. | 10.1186/s12879-022-07350-1 | Ethiopia | Associated | Not investigated | Not investigated | Measles non-vaccination among children aged 12-23 months | Children who were born from mothers who had birth orders one to three were four times more likely to drop out of the measles vaccine compared to those children who were born from mothers without/no birth order |
| Lakew, Y. | 10.11604/pamj.2015.21.208.7258 | Ethiopia | Associated | Not investigated | Not investigated | Full vaccination among children aged 12-23 months | Higher prevalence of full vaccination in the birth order 1 than others |
| Marefiaw TA,  Yenesew, MA,  Mihirete KM | 10.1371/journal.pone.0218470 | Ethiopia | Associated | Not investigated | Not investigated | Age-inappropriate pentavalent vaccination among children aged 12-23 months | Age-inappropriate pentavalent 1-3 vaccinations was associated decreasing birth order (AOR: 0.34, 95% CI: 0.17-0.68). |
| Hajizadeh M | 10.1016/j.vaccine.2019.09.054 | Gambia and Namibia | Associated | Not investigated | Not investigated | Full routine vaccination among children aged 0-59 months | Compared to children with birth order one, children with a later birth order had higher probabilities to follow the recommended child vaccination in the Gambia and Namibia. |
| Bettampadi D,  Carlson BF,  Mathew JL | 10.1016/j.amepre.2020.10.001 | India | Associated | Not investigated | Not investigated | Full vaccination among children aged 12-24 months | The AORs for full vaccination in the 3 surveys, respectively, were 0.68, 0.71, and 0.88 for birth order >1 versus birth order. |
| Islam T,  Mandal S,  Chouhan P | 10.1080/21645515.2021.1977056 | India | Associated | Not investigated | Not investigated | Full vaccination among children aged 12-23 months | Later birth order is associated with lower full immunization. |
| Nath B,  Singh JV,  Awasthi S,  Bhushan V, Kumar V, Singh SK | (..) | India | Associated | Not investigated | Not investigated | Full vaccination among children aged 12-23 months | Later birth order (OR 2) were found to have significant independent association with partial immunization. |
| Taneja G,  Datta E,  Sapru M,  Johri M,  Singh K,  Jandu HS et al. | 10.7759/cureus.35404 | India | Associated | Not investigated | Unadjusted prevalence | Zero-dose among children aged 12-25 months | The lowest prevalence of Zero-dose was among older children (birth order 1) than youngest children (6+). |
| Herliana P,  Douiri A | 10.1136/bmjopen-2016-015790 | Indonesia | Associated | Associated | Yes, both associated | Low immunization coverage among children aged 12-59 months | As the birth order increases, the likelihood of a child being unimmunised increases. |
| Siramaneerat I, Agushybana F | 10.22605/RRH6348 | Indonesia | Associated | Not investigated | Not investigated | Full vaccination among children aged 12-24 months | Children of second birth order (OR=1.934) had a 93.4% higher likelihood of receiving complete immunization than the first child. |
| Malderen CV, Ogali I, Khasakhala A, Muchirin SN, Sparks C,  Van Oyen H et al. | (..) | Kenya | Associated | Not investigated | Not investigated | Measles immunisation among children aged 12-24 months | Increasing a birth order category, decrease measles immunization. |
| Allan S, Adetifa IMO, Abbas K | 10.1186/s12879-021-06271-9 | Kenya | Associated | Not investigated | Not investigated | Full immunization coverage among children aged 12-23 months | The study showed a higher proportion of first-born child vaccinated compared to the sixth or later child born. |
| Mamuti S, Tabu C, Marete I, Opili D, Jalang’o R,  Abade A | 10.1371/journal.pone.0263780 | Kenya | Associated | Not investigated | Not investigated | MCV uptake among children aged 24-59 months | Children whose birth order was ≤5th born (POR = 0.46, 95% CI 0.22–0.95) were significantly associated with MCV uptake at the multivariate analysis |
| Fatiregun AA, Adebowale AS, Ayoka RO, Fagbamigbe AF | 10.1093/trstmh/trt079 | Nigeria | Associated | Not associated | Only birth order associated | Full vaccination among children aged 0-59 months | Being the older child (birth order 1) was risk factor for full vaccination compared to >=2. |
| Oleribe O, Kumar V, Awosika-Olumo A, Taylor-Robinson SD | 10.11604/pamj.2017.26.220.11453 | Nigeria | Associated | Not investigated | Not investigated | Full vaccination among children aged 0-59 months | There is an association between  child individual factors (child's sex and birth order). |
| Singh PK, Parsuraman S | (..) | Pakistan and India | Not investigated | Associated | Not investigated | Full immunization among children aged 12-23 months | Prevalence of child immunization was lower in female+1brother+1 sister (India) and female +2 sisters(Pakistan). Crude estimate. Results showed that in case of male older surviving sibling composition, the coverage of full immunization was lower in female children than in male children. |
| Bondya JN, Thinda A, Koval JJ, Speechleya KN | 10.1016/j.vaccine.2008.08.042 | Philippines | Associated | Associated | Not investigated | Full vaccination among children aged 12-23 months | Children who have  gone on to become fully immunized (as opposed to children who remain partially immunized) are more likely to have a lower birth order (i.e. eldest versus youngest), and to live in households with fewer children under the age of 5 years. |
| Mbengue MAS, Mboup A, Ly ID, Faye A, Camara FBN, Thiam M | (..) | Senegal | Associated | Not investigated | Not investigated | Untimely vaccination (BCG, polio, Penta3, MCV) among children aged 12-23 months | Associated with polio3 and penta3 - later birth order, higher risk of untimely vaccination). |
| Feldstein LF, Sutton R, Jalloh MF , Parmley L , Lahuerta M, Akinjeji A et al. | 10.7189/jogh.10.010420 | Sierra Leone | Associated | Not investigated | Not investigated | Incomplete pentavalent vaccine coverage among children aged 12-23 months | Children with the second or later birth order had 4.5 times higher odds of having incomplete pentavalent vaccine coverage. |
| Mohamud MH, Magan MA, Mohamed LM. Mohamud MA, Muse AA | 10.4103/jfmpc.jfmpc_119_20 | Somalia | Associated | Not investigated | Not investigated | Full vaccination among children aged 12-59 months | Young children are more likely to be fully vaccinated than older children. |
| Roy D, Debnath A, Sarma M, Roy D, Das K | 10.4103/ijcm.ijcm_422_22 | Southern Assam/India | Associated | Not investigated | Not investigated | Full vaccination among children aged 12-23 months | Children of a later birth order had a lower probability of receiving full vaccination compared to children of lower birth order. |
| Thang NM, Bhushan I, Bloom E, Bonu S | 10.1017/S0021932006001234 | Vietnam | Not associated | Not investigated | Not investigated | Zero-dose among children aged 11-23 months | There was no evidence of association between birth order and the rate of either fully immunized children or children having no immunization. |
| Marks JS, Halpin TJ, Irvin JJ, Johnson DA, Keller JR | (..) |  | Not investigated | Associated | Not investigated | Failure to complete his immunization among children aged 24 months | Completion of the basic series were increased in smaller family size (P less than .01). |

**(..)** DOI not available

**Supplemental Figure 1.** Ecological analyses of average birth order and average number of siblings, with 85 countries as the units of analysis. Pearson’s correlation coefficient = 0.994 (95% CI 0.990 to 0.996; p-value<0.001).

**Supplemental Figure 2.** Ecological analyses of zero-dose prevalence according to national gross domestic product per capita (GDP), with 85 countries as the units of analysis. Pearson’s correlation coefficient = -0.332 (95% CI -0.509 to -0.128; p-value<0.001).

**Supplemental Figure 3.** Ecological analyses of zero-dose prevalence according to national mean number of siblings, with 85 countries as the units of analysis. Pearson’s correlation coefficient = 0.506 (95% CI 0.328 to 0.649; p-value<0.001).

**Supplemental Figure 4.** Ecological analyses of zero-dose prevalence according to national mean number of siblings, with 85 countries as the units of analysis. *Note: The bars represent the prevalence of zero-dose and the whiskers the 95% Confidence Interval.*


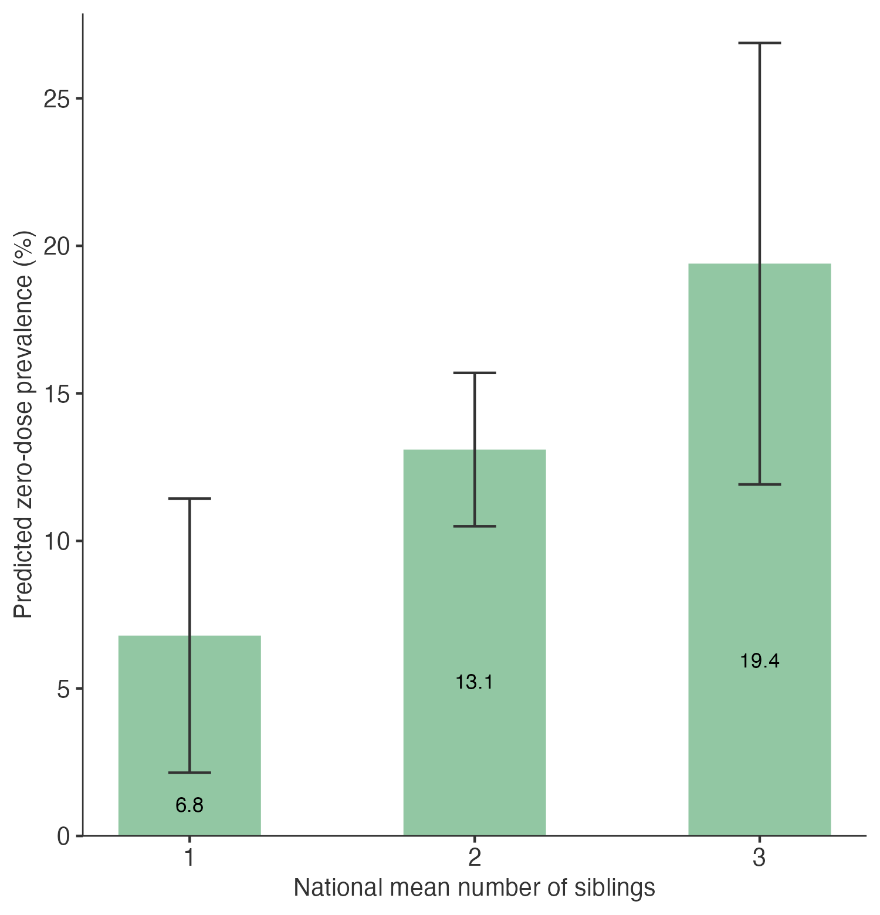


**Supplemental Figure 5.** Ecological analyses of zero-dose prevalence according to national mean birth order, with 85 countries as the units of analysis. Pearson’s correlation coefficient = 0.496 (95% CI 0.316 to 0.641; p-value<0.001).

**Supplemental Figure 6.** Ecological analyses of zero-dose prevalence according to national mean birth order, with 85 countries as the units of analysis. *Note: The bars represent the prevalence of zero-dose and the whiskers the 95% Confidence Interval.*


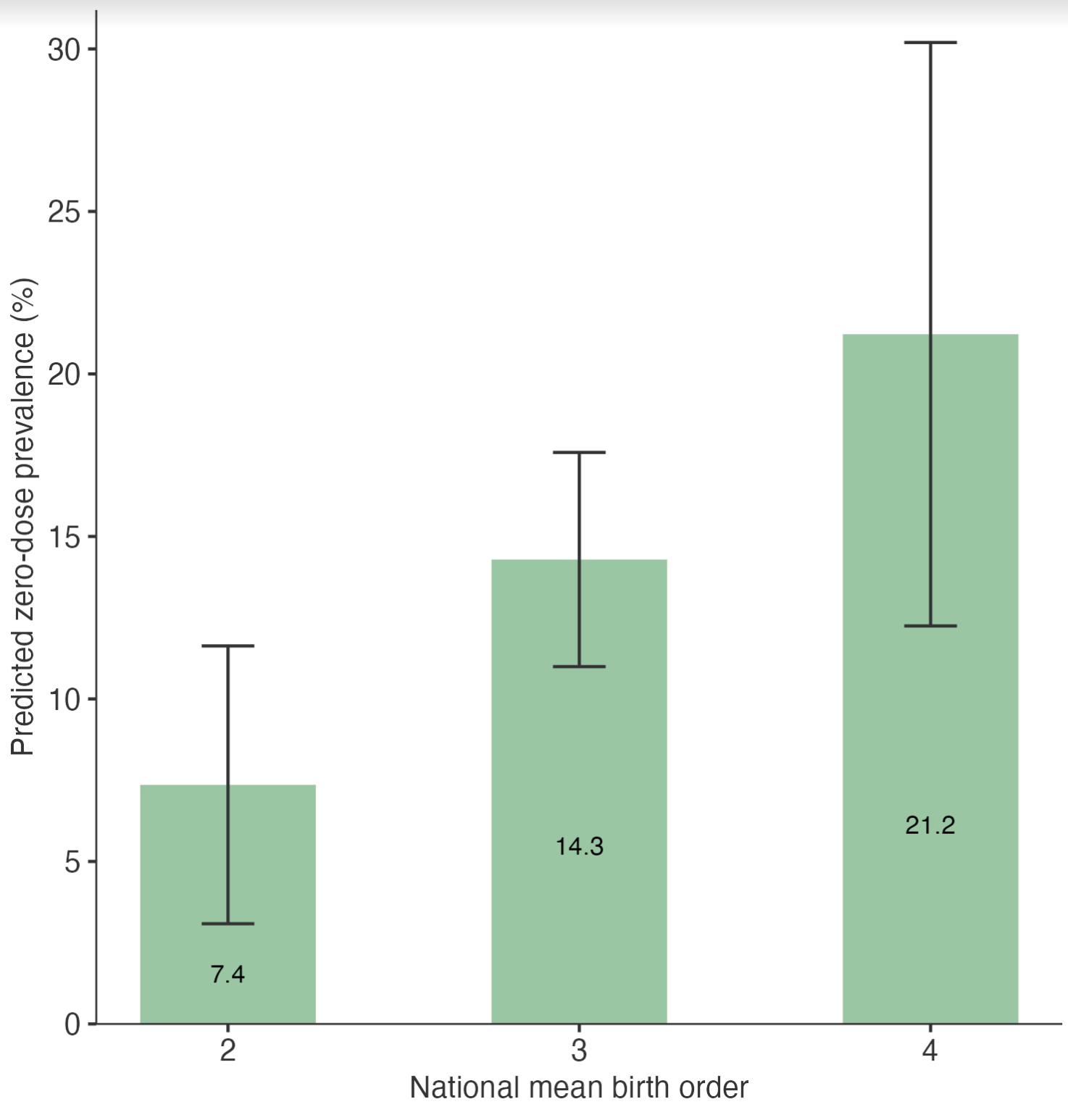

Supplement: Supplementary materials [file mmc1.docx]
